# Detection of Bovine *TMEM95* p.Cys161X Mutation in 13 Chinese Indigenous Cattle Breeds

**DOI:** 10.3390/ani9070444

**Published:** 2019-07-16

**Authors:** Sihuan Zhang, Kun Peng, Guoliang Zhang, Yang Cao, Meng Zhang, Hong Chen, Chuzhao Lei, Xianyong Lan, Yumin Zhao

**Affiliations:** 1 Branch of Animal Husbandry, Jilin Academy of Agricultural Sciences, Changchun 130033, China; 2 Key Laboratory of Beef Cattle Genetics and Breeding in Ministry of Agriculture and Rural Affairs, Changchun 130033, China; 3 Key Laboratory of Animal Genetics, Breeding and Reproduction of Shaanxi Province, College of Animal Science and Technology, Northwest A&F University, Yangling 712100, China

**Keywords:** Chinese indigenous cattle, molecular breeding, transmembrane protein 95 gene (*TMEM95*), male reproductive performance

## Abstract

**Simple Summary:**

Chinese indigenous cattle are economically important cattle breeds for animal husbandry development. The promotion and development of Chinese cattle breeds is essential. A previous study found that a nonsense mutation (rs378652941, c.483C > A, p.Cys161X) in bovine transmembrane protein 95 gene (*TMEM95*) seriously reduced reproductive performance in male Fleckvieh cattle; therefore, this locus was considered a candidate genetic marker in bovine marker-assisted selection (MAS) breeding. Until now, no study has identified this mutation in Chinese cattle breeds. Herein, we detected this c.483C > A mutation in 13 Chinese cattle breeds. Importantly, we found that this mutation did not exist at this locus in our analyzed breeds. Interestingly, we first identified a frameshift insertion/deletion (indel) mutation (NC_037346.1: g.27056998_27057000delCT) in the bovine *TMEM95* gene in 11 cattle breeds. Together, the results of this study suggest that the mutation c.483C > A cannot serve as a genetic marker for molecular breeding among Chinese indigenous cattle breeds.

**Abstract:**

Chinese indigenous cattle breeds have abundant genetic resources, which are valuable for the molecular breeding of cattle around the world. Thus, identifying important candidate genes and their genetic markers is very important for cattle molecular breeding. A previous study found that a nonsense mutation (rs378652941, c.483C > A, p.Cys161X) in the bovine transmembrane protein 95 gene (*TMEM95*) seriously reduced the reproductive performance in bulls, but few studies have detected this mutation in Chinese indigenous cattle breeds. Since the mutation c.483C > A may serve as a potential genetic marker for selecting higher fertility bulls, in the present study, using tetra-primer amplification refractory mutation system PCR (T-ARMS-PCR), forced PCR restriction fragment length polymorphism (forced PCR-RFLP), and DNA sequencing methods, the mutation c.483C > A was detected in 765 individuals from 13 Chinese cattle breeds. However, the results showed that this mutation did not exist at this locus in our analyzed breeds. Interestingly, we identified a newly frameshift insertion/deletion (indel) mutation (NC_037346.1: g.27056998_27057000delCT) in the bovine *TMEM95* gene in 11 cattle breeds, which changed the location of the termination codon and changed the 16 amino acids in the C-terminal to 21 amino acids. Combined with previous studies, our study provides evidence that in Chinese cattle breeds the mutation c.483C > A cannot be used as a genetic marker in molecular breeding.

## 1. Introduction

Cattle are among the most important domestic animals. China, vast in size and diverse in topography, has abundant cattle breeding resources. East Asian cattle are mainly composed of three distinct ancestries: East Asian taurine ancestry, Eurasian taurine ancestry, and Chinese indicine ancestry [1]. The earlier East Asian taurine ancestry reached China at least 3.9 thousand years ago [1]. China’s abundant cattle breeding resources are helpful for breeding new species or improving the reproductive performance of cattle around the world. Traditional crossbreeding depends on appearance identification and phenotypic selection, which are costly and time-consuming [2]. Nowadays, molecular breeding has brought about great changes in the field of animal breeding, such as marker-assisted selection (MAS) breeding [3]. MAS breeding is based on the functional genes or molecular markers, thus identifying genes or genetic variations is essential to the breeding of cattle [3].

Artificial insemination (AI) is a routine breeding technique in cattle production. The application of AI in cattle began at the beginning of the 20th century, and improves the breeding efficiency of bulls [4,5]. In order to improve production efficiency, only bulls with excellent reproduction performance can be used as sires to provide high-quality semen. However, the success rate of AI varies from sire to sire [6]. This phenomenon suggests that there are some factors that affect fertilization rates without affecting sperm quality [7]. In 2014, Pausch et al. found a nonsense mutation (rs378652941, c.483C > A, p.Cys161X) in cattle transmembrane protein 95 gene (*TMEM95*) gene was associated with idiopathic male subfertility in Fleckvieh cattle, but did not affect semen quality [7]. Subfertility refers to any form of reduced fertility, which causes enormous economic losses in animal husbandry [7,8]. Bovine *TMEM95* consists of 183 amino acids, encoding a quite conserved single-pass type I transmembrane protein (1 to 16 form a predicted extracellular signal peptide; 17 to 152 form the extracellular sequence; 153 to 175 form the transmembrane domain; 176 to 183 form the intracellular C-terminal domain) [7]. c.483C > A was identified in the transmembrane domain, introducing a premature stop-codon in *TMEM95* (p.Cys161X), which truncates 22 amino acids of the *TMEM95* C-terminal sequence [7]. A functional study found that TMEM95 protein localizes on the acrosomal membrane of the sperm head, and c.483C > A most likely results in disturbed anchorage of the truncated protein in the sperm plasma membrane bilayer [7]. Recently, Fernandez-Fuertes et al. revealed that c.483C > A reduced fertility by influencing the interaction with the oocyte vestments [9,10,11]. These studies indicated that c.483C > A is a deleterious mutation that should be identified and eliminated as early as possible in cattle.

Until now, no study has detected the abovementioned nonsense mutation (c.483C > A) in Chinese cattle. Considering the genetic effect of bovine *TMEM95* gene mutation on male reproductive performance, testing and verifying the candidate molecular genetic markers in Chinese cattle breeds is beneficial and necessary. Herein, a total of 765 individuals from 13 Chinese representative cattle breeds were selected for this study, so as to lay the foundation for assessing the genetic variations of the bovine *TMEM95* gene and evaluating the possibility of applying the genetic markers in cattle MAS breeding.

## 2. Materials and Methods 

All animal experiments in the present study were performed in conformity to the applicable guidelines, animal welfare laws, and policies by the Ministry of Science and Technology of the People’s Republic of China (Approval Number 2006-398). Moreover, the experiments were also approved by Northwest A&F University (NWAFU), Shaanxi, P.R. China.

### 2.1. Animal Collection and Genomic DNA Isolation

A total of 765 cattle samples from 13 Chinese indigenous cattle breeds were analyzed: Red Steppe cattle (*n* = 135, Jilin Province), Qinchuan cattle (*n* = 60, Shaanxi Province), Nanyang cattle (*n* = 60, Henan Province), Jinnan cattle (*n* = 60, Shanxi Province), Luxi cattle (*n* = 30, Shandong Province), Xia’nan cattle (*n* = 60, Henan Province), Jiaxian Red cattle (*n* = 60, Henan Province), Pi’nan cattle (*n* = 60, Henan Province), Jinjiang cattle (*n* = 60, Jiangxi Province), De’nan cattle (*n* = 30, Henan Province), Yunling cattle (*n* = 60, Yunnan Province), Zaosheng cattle (*n* = 30, Gansu Province), and Bohai Black cattle (*n* = 60, Shandong Province).

All selected individuals were unrelated and healthy. By means of the phenol-chloroform method, the genomic DNA was isolated from ear tissue [12,13]. The quality of genomic DNA sample was assayed by a NanoDrop 2000 Spectrophotometer (Thermo Scientific, Waltham, MA, USA). DNA samples were then diluted to the identical standard of 50 ng/µL and stored at −20 °C [12,13].

### 2.2. Primer Design and Genotyping by T-ARMS-PCR and Forced PCR-RFLP Methods

A previous study found a potential nonsense mutation (rs378652941, c.483C > A, p.Cys161X) in bovine *TMEM95*, which introduced a premature stop-codon in the *TMEM95* gene [7]. Based on the GenBank sequence of the bovine *TMEM95* gene (NC_037346.1), the primers of tetra-primer amplification refractory mutation system PCR (T-ARMS-PCR), which include two allele-specific inner primers and a pair of outer primers, were designed using the Primer1 website (http://primer1.soton.ac.uk/primer1.html) [14,15]. The inner primer contained a second deliberate mismatch at position 2 at the 3′ end. The principle of the T-ARMS-PCR method is that Taq DNA polymerase lacks the 3′ → 5′ exonuclease activity, and if the primer’s 3′ end is mismatched then its amplification rate is slower. With the use of specific inner and outer primers at the exact proportion and amplification conditions, different alleles of the locus are amplified. The amplification product, which includes two homozygous and three heterozygous bands, is detected by gel electrophoresis [16]. The primers of forced PCR restriction fragment length polymorphism (forced PCR-RFLP) and the primers used for PCR amplification and sequencing were designed with Primer Premier software (version 5.0) (PREMIER Biosoft International, Palo Alto, CA) (Table 1). The forced PCR-RFLP was based on the PCR-RFLP method. Because the mutation locus could not be recognized by commonly used restriction enzymes, we designed a primer to change a base near the mutation site to induce a restriction enzyme recognition sequence. Then the mutation could be genotyped according to the bands of the PCR products digested by restriction enzymes [16].

The PCR reaction was carried out in a 13 μL reaction volume, and the PCR programs was the same as in our previous studies [17]. Using 3.0% agarose gel, the T-ARMS-PCR product and forced PCR-RFLP product were examined by electrophoresis. Using the T-ARMS-PCR program, the length of the amplified fragments would be 312 bp (outer)/196 bp (C)/168 bp (A). The product lengths of the forced PCR-RFLP amplification digested by *Hha* I endonuclease were 228 bp and 25 bp for the CC genotype; 253 bp, 228 bp, and 25 bp bands for genotype AC; genotype AA showed one band of 253 bp. The genotypes of individuals were classified by analyzing the electrophoresis images.

### 2.3. Genomic DNA Pool Construction and DNA Sequencing 

**Figure 1:**
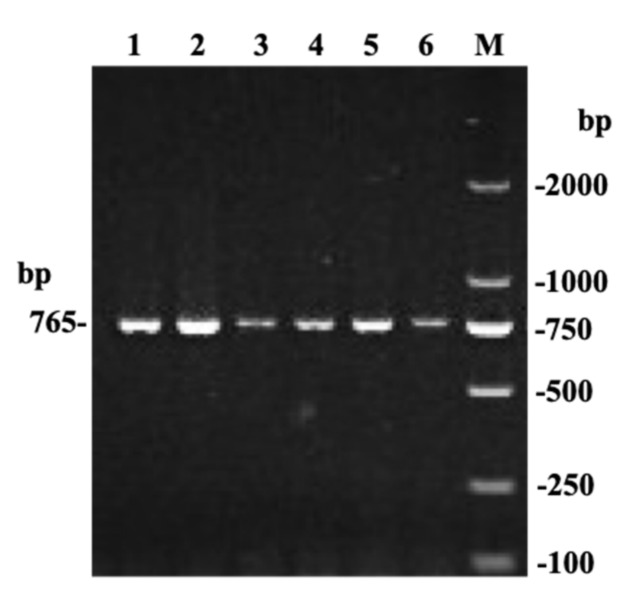
Agarose gel electrophoresis pattern of the bovine *TMEM95* gene.

**Note:** 765 bp bands represent the target sequencing products, (Lane Marker) DNA Marker D2000; Lanes 1–6 are the products of six DNA pools among 13 Chinese indigenous cattle breeds.

This study also verified the genotypes by sequencing; the PCR product for sequencing is 765 bp in length. Red Steppe cattle were sequenced one by one. Then, we constructed 21 DNA pools in the analyzed cattle breeds (for the following breeds, two DNA pools were constructed for each breed: Pi’nan cattle, Nanyang cattle, Jiaxian Red cattle, Xia’nan cattle, Jinnan cattle, Bohai Black cattle, Qinchuan cattle, Yunling cattle, and Jinjiang cattle; for the following breeds, one DNA pool was constructed for each breed: De’nan cattle, Zaosheng cattle, and Luxi cattle). In each DNA pool, 30 DNA samples were randomly selected, then the DNA pools or DNA were used for amplifications and sequencing. The PCR reaction was performed in a 25 μL volume, and touchdown PCR reaction was implemented according to six steps, as follows [18,19]: step 1: initial denaturation at 95 °C (5 min); step 2: 17 cycles of denaturation at 94 °C (30 s); step 3: annealing at 68 °C (30 s), with a decrease of 1 °C per cycle; step 4: extension for 1000 bp/min at 72 °C; step 5: another 30 cycles at 94 °C (30 s), at 51 °C (30 s), and at 72 °C (1000 bp/min); step 6: a final extension at 72 °C (5 min). When the PCR amplification product was the objective band, it was sequenced to screen the target c.483C > A locus (Figure 1). 

## 3. Results

### 3.1. SNP Identification and Genotyping of the Bovine *TMEM95* Gene by T-ARMS-PCR and Forced PCR-RFLP

In a previous study [7], the nonsense mutation (rs378652941, c.483C > A, p.Cys161X) was identified in the male Fleckvieh cattle *TMEM95* gene. In this study, we failed to detect the different genotypes of this locus via the T-ARMS-PCR and forced PCR-RFLP methods. 

### 3.2. Sequencing Results of the *TMEM95* Gene in 13 Chinese Indigenous Cattle Breeds

**Figure 2:**
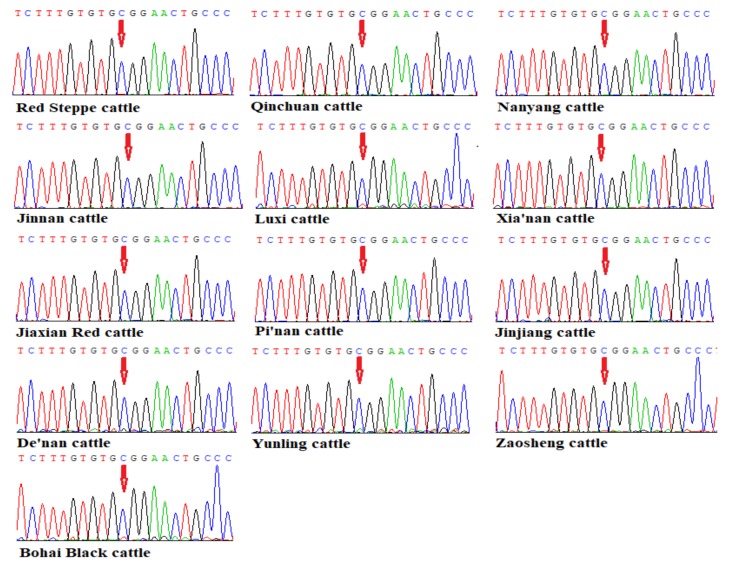
The sequencing results of bovine *TMEM95* c.483C > A mutation in 13 different cattle breeds.

We further used the DNA sequencing method to identify the genotypes of this locus [20]. First, we sequenced the Red Steppe cattle PCR products one by one, but no mutation was found in the c.483C > A locus. Then, we constructed genomic DNA pools to identify the mutation in the other cattle breeds. The sequencing results showed that there was no mutation in our analyzed 13 Chinese cattle breeds in the c.483C > A locus, and all the individuals were CC genotype (Table 2 and Figure 2). 

**Figure 3:**
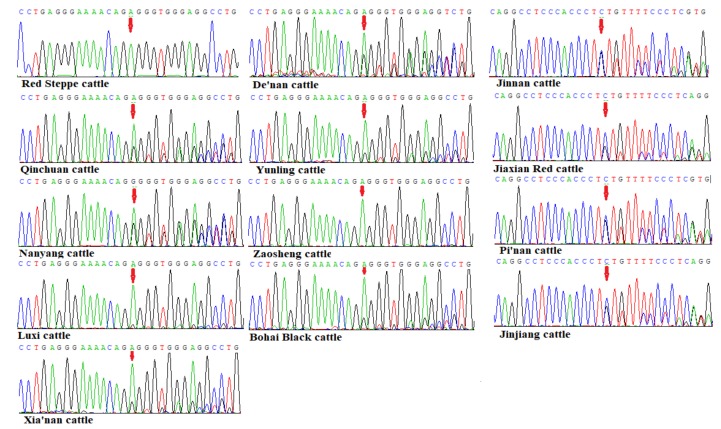
The sequencing results of bovine *TMEM95* indel mutation (NC_037346.1: g.27056998_27057000delCT) in 13 different cattle breeds.

Interestingly, by amplifying and sequencing analyses of the 765 bp PCR products, a novel 2 bp indel mutation in the bovine *TMEM95* gene, named NC_037346.1: g.27056998_27057000delCT, was revealed in the detected cattle breeds. This indel was found in 11 studied cattle breeds (Nanyang cattle, Jiaxian Red cattle, Xia’nan cattle, Jinnan cattle, Bohai Black cattle, Qinchuan cattle, Yunling cattle, Pi’nan cattle, Jinjiang cattle, De’nan cattle, and Luxi cattle). However, in Zaosheng cattle and Red Steppe cattle no mutation was revealed in this indel locus (Figure 3). This 2 bp indel was a frameshift mutation, which changed the location of the termination codon and changed the 16 amino acids in the C-terminal to 21 amino acids.

## 4. Discussion

In 2014, the nonsense mutation c.483C > A (p.Cys161X) in the bovine *TMEM95* gene was first detected in the male Fleckvieh cattle population by Pausch et al. [7]. In 2017, Fernandez-Fuertes et al. [10] examined the effects of this mutation on bovine sperm function. Their findings showed that in vivo and in vitro the male reproductive performance of the CC and CA genotypes is normal, but the AA genotype exhibits extremely poor fertility. Based on these findings, it is necessary to scientifically identify this genetic variant in more cattle breeds as potential genetic markers for selecting bulls with better reproductive performance.

In the present study, we first detected the c.483C > A in 13 Chinese indigenous cattle breeds. Surprisingly, there was no mutation in the c.483C > A locus in the detected Chinese cattle breeds. So, the fact that the nonsense mutation c.483C > A only exists in Fleckvieh cattle but not in Chinese cattle breeds (at least in the breeds analyzed in this study) suggests that this locus is not suitable to be used in MAS breeding in Chinese cattle. Still, in order to understand why Chinese cattle breeds did not contain this mutation, follow-up studies are necessary to clarify the breeding discrepancy and reproduction differences between foreign and Chinese cattle breeds. 

In this study, an indel mutation (NC_037346.1: g.27056998_27057000delCT) was discovered in 11 cattle breeds by direct sequencing of the bovine *TMEM95* gene. This is the first study to detect the indel mutation in this gene. Multiple studies have suggested that indel mutations located in coding or non-coding regions have notable effects on gene expression and phenotypes [21]; thus, this mutation might significantly affect the function of bovine *TMEM95*, though that needs further verification. Furthermore, two alternative splicing variants of the *TMEM95* gene have been identified in the brain and testicular tissue of fetal Qinchuan cattle [22]. Alternative splicing plays a critical role in regulating biological function and is viewed as a crucial factor in generating functional diversity proteins in metazoan organisms [23]. Taken together, this study indicates that the bovine *TMEM95* gene has abundant genetic polymorphisms, but further investigation is needed to better understand the function of this gene as well as enhance the male reproductive performance of bulls.

## 5. Conclusions

In this study, T-ARMS-PCR, forced PCR-RFLP, and DNA sequencing methods were used to detect the c.483C > A mutation in 765 individuals from 13 Chinese cattle breeds. The c.483C > A mutation was not found in our analyzed breeds. However, we identified a new frameshift indel mutation (NC_037346.1: g.27056998_27057000delCT) in the bovine *TMEM95* gene in 11 cattle breeds, which changed the location of the termination codon and changed the 16 amino acids in the C-terminal to 21 amino acids. These results suggest that the c.483C > A mutation cannot serve as a genetic marker in molecular breeding in Chinese indigenous cattle breeds.

## Figures and Tables

**Table 1:** The primers for bovine *TMEM95* c.483C > A mutation identification.

Methods	Primers (5′-3′)	^a^ Regions	Genotype Pattern (bp)
T-ARMS-PCR	F(outer): CCTCACCCCCACCCAGATCTCTGAGCTC (1731-1758)	Intron 5 to Exon 6	Product of outer primer = 312 CC = 312 + 196; CA = 312 + 196 + 168; AA = 312 + 168.
	R(outer): ACCTGAGGGAAAACAGAGGGTGGGAGGC (2015-2042)		
	F(inner A): CTCGGATCCTGCTCCTCTTTGTGCGC (1847-1872)		
	R(inner C): GGGACACCCAGGAGCAGGGCAGTTTCT (1872-1898)		
Forced PCR-RFLP	F: AAGCTCGGATCCTGCTCCTCTTTGTGCG (1844-1869)	Exon 6	*Hha* I (GCG↓C) AA = 253; AC = 253 + 228 + 25; CC = 228 + 25.
	R: GGCTAGGCTCTGTCCTCGTTT (2076-2096)		
Sequencing	F: GTGAGTAAGAAAGGGAAGGGGTCG (1498-1519)	Exon 4 to Exon 6	765
	R: ACCATCTGACACTGGGACTA (2243-2262)		

Note: The c.483C > A mutation was identified on the 1872nd nucleic acid of bovine *TMEM95* gene (NCBI Reference Sequence: NC_037346.1). ^a^ The regions relative to the bovine *TMEM95* gene mRNA sequence (XM_010815969.3). T-ARMS-PCR: tetra-primer amplification refractory mutation system PCR; PCR-RFLP: PCR restriction fragment length polymorphism.

**Table 2:** Genotypic and allelic frequencies of bovine *TMEM95* c.483C > A (p.Cys161X) mutation.in 13 Chinese indigenous cattle breeds.

Breeds	Total (*n*)	Allelic Frequency (%)		Genotypic Frequency (%)		
		Cys	X	Cys/Cys	Cys/X	X/X
Red Steppe cattle	135	270 (100)	0 (0)	135 (100)	0 (0)	0 (0)
Qinchuan cattle	60	120 (100)	0 (0)	60 (100)	0 (0)	0 (0)
Nanyang cattle	60	120 (100)	0 (0)	60 (100)	0 (0)	0 (0)
Jinnan cattle	60	120 (100)	0 (0)	60 (100)	0 (0)	0 (0)
Luxi cattle	30	60 (100)	0 (0)	30 (100)	0 (0)	0 (0)
Xia’nan cattle	60	120 (100)	0 (0)	60 (100)	0 (0)	0 (0)
Jiaxian Red cattle	60	120 (100)	0 (0)	60 (100)	0 (0)	0 (0)
Pi’nan cattle	60	120 (100)	0 (0)	60 (100)	0 (0)	0 (0)
Jinjiang cattle	60	120 (100)	0 (0)	60 (100)	0 (0)	0 (0)
De’nan cattle	30	60 (100)	0 (0)	30 (100)	0 (0)	0 (0)
Yunling cattle	60	120 (100)	0 (0)	60 (100)	0 (0)	0 (0)
Zaosheng cattle	30	60 (100)	0 (0)	30 (100)	0 (0)	0 (0)
Bohai Black cattle	60	120 (100)	0 (0)	60 (100)	0 (0)	0 (0)
**Total**	**765**	**1530 (100)**	**0 (0)**	**765 (100)**	**0 (0)**	**0 (0)**
